# Microbiota, Bacterial Carbonic Anhydrases, and Modulators of Their Activity: Links to Human Diseases?

**DOI:** 10.1155/2021/6926082

**Published:** 2021-11-11

**Authors:** Amedeo Amedei, Clemente Capasso, Giulia Nannini, Claudiu T. Supuran

**Affiliations:** ^1^Department of Experimental and Clinical Medicine, University of Florence, 50134 Florence, Italy; ^2^SOD of Interdisciplinary Internal Medicine, Azienda Ospedaliera Universitaria Careggi (AOUC), 50134 Florence, Italy; ^3^CNR, Institute of Biosciences and Bioresources, 80131 Napoli, Italy; ^4^Department of Neurofarba, University of Florence, Florence, Italy

## Abstract

The involvement of the human microbiome is crucial for different host functions such as protection, metabolism, reproduction, and especially immunity. However, both endogenous and exogenous factors can affect the balance of the microbiota, creating a state of dysbiosis, which can start various gastrointestinal or systemic diseases. The challenge of future medicine is to remodel the intestinal microbiota to bring it back to healthy equilibrium (eubiosis) and, thus, counteract its negative role in the diseases' onset. The shaping of the microbiota is currently practiced in different ways ranging from diet (or use of prebiotics, probiotics, and synbiotics) to phage therapy and antibiotics, including microbiota fecal transplantation. Furthermore, because microbiota modulation is a capillary process, and because many microbiota bacteria (both beneficial and pathogenic) have carbonic anhydrases (specifically the four classes *α*, *β*, *γ*, and *ι*), we believe that the use of CA inhibitors and activators can open up new therapeutic strategies for many diseases associated with microbial dysbiosis, such as the various gastrointestinal disorders and the same colorectal cancer.

## 1. Introduction

The term microbiome refers to the whole habitat, including the different microorganisms (bacteria, archaea, eukaryotes, and viruses) that define the so-called microbiota, their genomes, and the environmental conditions. Lederberg and McCray first specified the expression microbiota, who pointed out the importance of microorganisms inhabiting the human body in health and disease [1]. However, we have only recently started to recognize that the human body is home to much more than human cells [2]: we shelter at least 100 trillion (10^14^) microbial cells and a quadrillion viruses [3]. As said before, this intricate community includes bacteria, eukaryotes, viruses, and at least one archaeon that interact with each other and with the host, resulting in a significant impact on human health and physiology. Only a tiny portion of these can be cultured, and high-throughput sequencing has recently seriously increased the range of known microbes in our bodies and the environment [2, 4]. The gut microbiota (GM) composition mirrors the natural selection at both microbial and host levels, fostering mutual interplay and functional stability of this complex ecosystem [5]. Acid and pancreatic secretions usually prevent bacterial colonization of the stomach and proximal small intestine. Bacterial density, however, rises in the distal small intestine and increases to an estimated 10^11^-10^12^ bacteria per gram of colonic matter in the large intestine, contributing to 60% of the fecal mass [5, 6]. The foetal gut seems sterile, but colonization starts immediately after birth and is affected by the delivery mode, infant diet, hygiene levels, and antibiotic intake [7]. In the environment, humans coevolved with microbes, and each body habitat has in its microbiota a unique set of microorganisms that are established in the first 1-3 years of life and remain relatively stable over the entire life span [8]. The microbiota was generally characterized using molecular methods primarily based on the analysis of 16S rRNA genes or other marker genes and genomic regions, amplified and sequenced from the biological samples provided [9]. Several tools that assign each sequence to a microbial taxon (bacteria, archaea, or lower eukaryotes) may be used to perform taxonomic assignments at different taxonomic levels according to phyla, groups, orders, families, genera, and organisms [9]. Just a few phyla are represented in each body district, accounting for hundreds of species of bacteria [10].

For body physiology, the human microbiome is crucial, producing an enormous number of molecules able to communicate with the host. In particular, gut bacteria are a natural protection against pathogens, and, in addition, they break down indigestible dietary components (e.g., vegetal polysaccharides) [11]. The metabolic functions of residential microbes are involved in host functions, such as protection, metabolism, reproduction, and immunity [12]. It consists of two predominant phyla, *Firmicutes* and *Bacteroidetes* (about 90% of the total bacteria), while the remaining 10% is split between *Verrucomicrobia*, *Proteobacteria*, and *Actinobacteria* [13]. Notably, if, on the one hand, the microbiota participates in the maturation of the host immunity and its functionality, on the other hand, it is modulated by the host's immune system [6]. The GM role is crucial for the proper development of the gut-associated lymphoid tissue (GALT) and the expected evolution of the innate and specific immune system [14]. The microbiota-immunity axis enables the optimal arrangement of the innate and adaptive immune response in eubiosis conditions to modulate the most suitable reaction [15]. The recent increase of microbiome studies sheds light on its contributing impact on etiology and the progression of many diseases. A microbiota imbalance, named “dysbiosis,” can cause significant effects on the host [16]. Understanding the link between illness and dysbiosis could let researchers sufficiently define the development of an increasing number of human diseases and discover innovative treatments, modulating the microbiota composition to restore its eubiosis status and so the host health.

## 2. Link between Dysbiosis and Pathologies

Gut dysbiosis has a drastic impact on gut health. As reported by the American Gastroenterologist Association's journal, Crohn's disease (CD), ulcerative colitis (UC), and pouchitis are the results of the pathogenic immune response following gut microbiota antigenic stimulation consequently to mucosal barrier defects [17]. Recently, we have described a dissimilarity of cytokines' distribution and microbiota composition within the CD and the adjacent healthy ileal tissue layers and, in addition, between the first operation and surgical relapse [18]. Another relevant disease deeply correlated with gut dysbiosis is Clostridioides difficile Infection (CDI), caused by opportunistic bacteria responsible for infectious colitis in hospitalized patients [19]. Considering that CDI occurs in patients with disrupted gut microbiota, it seems easy to hypothesize that healthy gut microbiota can prevent Clostridioides difficile colonization [20]; in fact, the fecal microbiota transplant can contrast the infection restoring a functional microbiota, as recently documented by a systematic study [21]. Microbiota composition in cancer biology has been increasingly accepted as an environmental factor favoring colorectal cancer (CRC) development. Microbial dysbiosis associated with CRC can alter the delicate balance between the gut microbiota and the host's immune system, leading to cancer initiation and/or progression [22]. As a result, CRC can be avoided by converting the microbiome to a noncarcinogenic microbiome. In this context, probiotics are being explored for their potential function in CRC prevention and treatment and as an adjunct to conventional therapy [23]. The role of *Fusobacterium nucleatum* is very emblematic. It promotes CRC by the induction of epithelial cell proliferation [24], enabling a proinflammatory microenvironment [25] and producing proteins able to stop the antitumoral activity of T and NK cells [26, 27]. The data of our recent study [28] suggest that microbial communities can drive and modulate the antitumor immune response. We have shown for the first time that in CRC, *Prevotell*a and *Bacteroides* species are correlated positively and negatively, respectively, with the secretion of IL-9, which has a fascinating and still debated role in tumor immunity.

In the context of cancer, the microbiota is also involved in other tumor types. As previously reported, changes in microbiome composition seem to induce or exacerbate chronic inflammation, leading to immune surveillance disruption. Besides, concerning the intestinal microbiota, the link between local microbiota and some cancers has been elucidated, connecting local dysbiosis and carcinogenesis. Hayes et al. have demonstrated that increased relative abundance of *Corynebacterium* and *Kingella bacteria* in the oral microbiome has been associated with reduced incidence of oral cavity carcinomas [29]. Different gut microbiome composition has been shown among patients with pancreatic malignancies compared to healthy controls [30].

Finally, chronic inflammation, related to altered lung microbiota, could explain local carcinogenesis in lung cancer. It has been demonstrated that several bacteria species have been enriched in lung cancer patients compared with healthy individuals [31, 32]. Lung microbiota modifications induced by antibiotics could explain the higher incidence of lung cancer among users of antibiotics as reported in a recent meta-analysis [33].

Still, regarding cancer, the development of immune checkpoint inhibitors (ICIs) has transformed the therapeutic view for many malignancies. Several lines of evidence have indicated that the gut microbiome plays a crucial role in modulating immune checkpoint blockade response across a range of cancer types [34–36]. In responders to anti-PD1 antibody, a higher gut microbial alpha diversity was highlighted, as the relative abundance of the order Clostridiales, the *Ruminococcaceae* family, and the species *Faecalibacterium prauznitzii* [36]. Regarding other pathologies, Novakovic [37] and Oikonomou [38], in two recent reviews, analyzed some studies, focused on the interplay between microbiota-immune response with cardiovascular diseases, for which hypertension represents the leading risk factor. Several data [39, 40] have highlighted that atherosclerosis development, the dominant cause of cardiovascular diseases, is linked with trimethylamine N-oxide (TMAO) levels on that the changes in GM composition have marked effects.

The gut-brain-microbiome axis is one of the principal pathways for the interplay between the microbiome and disease. Although most studies are in preclinical stages, evidence suggests continuous communication along this axis. In particular, the brain responds to gut microbiome signals in order to change gut motility and permeability, influencing the microbiota functionality [41]. In this scenario, increasing studies focus on the link between the gut-brain-microbiome axis and neurodegenerative diseases, such as Parkinson's disease, Alzheimer's disease (AD), and amyotrophic lateral sclerosis (ALS). Marizzoni and his group [42] reported that gut microbiota-related products, such as lipopolysaccharides and short-chain fatty acids, and systemic inflammation are related to brain amyloidosis presence in older human subjects. In addition, a recent study by Mandrioli et al. [43] proposed a trial to evaluate the biological basis of a potential treatment for ALS using the FMT. In ALS, GM dysbiosis may favor the disease onset or drive its progression and related outcomes in the presence of other risk factors. Otherwise, ALS presence could further alter GM dysbiosis and, in some individuals, lend to disease progression and prognosis and affect treatment response [44].

## 3. Microbiota Shaping: Focus on the Antibiotic Therapy

The imbalance of the gut microbiota of the bacterial species has been demonstrated to be prevalent among various debilitating diseases. Diet, prebiotics, probiotics, symbiotics, FMT, phage therapy, and antibiotics are some of the new emerging therapeutic options. All of these are aimed at restoring gut homeostasis, microbiota composition, and physical barrier defense.

### 3.1. Diet

Diet is probably the most readily modifiable environmental factor, but few studies have accurately investigated the link between diet and GM composition [45–47]. Increasing evidence suggests that diets low in animal protein and high in vegetable and fiber intake are related to the prevention of cardiovascular disease [48]. A fascinating study of Pagliai et al. evaluated the functional composition of the fecal microbiota in a short-term, fully controlled low-calorie Mediterranean and vegetarian diet. They found that the short-term Mediterranean or vegetarian dietary pattern does not cause significant modification in the GM composition, implying that nutritional interventions should be sustained for more extended periods to scratch GM resilience [49].

### 3.2. Probiotics, Prebiotics, and Synbiotics

Probiotics are live microorganisms that, when given in sufficient amounts, provide health benefits to the host. Probiotics have been shown in multiple studies to be effective in alleviating diarrhea and other gut-related side effects associated with anticancer therapy, restoring a healthy GM composition [50]. In detail, *Bifidobacterium* spp., *Lactobacillus* spp., *Lactococcus* spp., *and Saccharomyces boulardii* are the most routinely employed probiotics [51]. Prebiotics include nondigestible polysaccharides and oligosaccharides, among which inulin, lactulose, fructooligosaccharides, and galactooligosaccharides that are fermented by colonic bacteria, resulting in specific changes in the GM composition and functions. Prebiotic fibers can be found in various foods, primarily in vegetables like asparagus, garlic, leeks, and onions [52]. Prebiotics promote the growth of protective bacteria in the intestine, especially *Bifidobacterium* and *Lactobacilli*, and reduce intestinal permeability and metabolic endotoxemia [53]. Finally, synbiotics are a blend of prebiotics and probiotics that can help the host.

### 3.3. Fecal Microbiota Transplant

The injection by different ways (via colonoscopy, enema, orogastric tube, or by mouth in the form of a capsule) of feces from a healthy donor into the gastrointestinal tract of a recipient patient is known as FMT [54]. As a result, there is a chance to restore the complexity and diversity of the intestinal microbiota, even though probiotics are beneficial [55]. As previously reported, FMT is effective in treating recurrent CDI, with a cure rate of about 90%. FMT tends to be healthy in a short-term follow-up, with the most common recorded side effects including abdominal pain, diarrhea, constipation, and low-grade fever. Long-term consequences could include the transmission of undiagnosed infections that can cause disease years later and lead to chronic diseases like obesity, diabetes, NAFLD, asthma, and autism, as reported in case reports [56, 57]. Recent studies suggest a potential FMT role in improving anticancer therapy efficacy and adverse events. This therapeutic role of FMT was shown with some chemotherapy agents, immunotherapy, and radiotherapy [58, 59].

### 3.4. Phage Therapy

Phages have been used as therapeutic instruments since their discovery more than a century ago. Despite their success in the first trials, the use of phage therapy was controversial and not generally accepted [60]. In the last few years, thanks to scientific progress in metagenomics and the consciousness of the intestinal microbiota importance to maintain human health, research on the intestinal phagome has brought up interest [61]. Phages have been mainly explored as promising tools in infectious diseases, among which cholera and Clostridioides difficile colitis and the eradication of adherent invasive Escherichia coli (AIEC) in Crohn's disease [62, 63]. The most critical aspect of phage therapeutic development is determining the phages' safety and efficacy. Currently, in vivo animal models or a suitable in vitro system is used in this research field [64].

### 3.5. Antibiotics

Antibiotics are a strong weapon against pathogenic bacteria but can also damage commensal organisms, leading to the loss of microbial diversity and reduced colonization resistance against pathogens [65]. A few days after antibiotic treatment, profound and swift modifications occur in the GM composition. Despite this, antibiotic therapy plays a fundamental role in microbiota manipulation. Small intestinal bacterial overgrowth (SIBO) is characterized by an abnormal number of bacteria in the small intestine and is followed by numerous gastrointestinal symptoms. The target in SIBO patients is to relieve symptoms through bacteria eradication [66], and antibiotics are widely used. However, some patients remain symptomatic after care, meaning that other underlying disorders and/or antibiotic-resistant bacteria are to blame [67]. Currently, rifaximin, a nonsystemic antibiotic, is the most studied drug for SIBO patients. Numerous studies demonstrated its efficacy, even if the dose, treatment duration, diagnostic methods, and patients vary among studies [68–73]. A meta-analysis of rifaximin (dose range: 600–1,600 mg/d; duration of treatment: 5–28 days) documented that SIBO was eradicated in 70.8% of patients (26 studies; 95% CI, 61.4–78.2). Adverse events were rare, occurring in just 4.6 percent of 815 patients in 17 studies that reported safety [74]. Also, systemic antibiotics as ciprofloxacin, norfloxacin, and metronidazole reported SIBO eradication with both the breath test or bacterial culture [75–78]. Antibiotic GM regulation was only partially investigated in CRC, with only a few reports in the literature [79]. Cefoxitin is a semisynthetic and broad-spectrum cephalosporin that induced a complete and lasting clearance of enterotoxigenic *B. fragilis* colonization in previously ETBF-inoculated mice with a reduction in median adenoma formation [80]. Erythromycin can suppress the transcriptional activity of NF-*κ*B, the activator protein-1 (AP-1), and its downstream targets, IL-6 and cyclooxygenase-2 (COX-2), in human CRC cells [81]. As reviewed by Elkrief et al., antibiotics with a wide action range harm the outcomes of patients receiving ICIs [82]. Nevertheless, there could be present specific antibiotics that might induce favorable alterations in the host immune system even if the problem of antibiotics with a spectrum narrow enough to ensure a fine depletion remains. Some patients, in fact, could show an abundance of species that promote immune suppression through the activation and expansion of the regulatory T cells (Tregs). A recent clinical trial highlights the depletion of vancomycin-sensitive bacteria resulting in boosted radiotherapy's antitumor immune response and inhibition of tumor growth [83].

## 4. Bacterial Carbonic Anhydrases and Their Modulation

As mentioned above, it is evident that (a) microbiota metabolism has a crucial impact on the human intestine, acting as a self-regulating system and influencing those districts known as gut-brain, gut-liver, gut-kidney, and gut-heart; (b) microbiota tunes negatively or positively the host health.

Here, we focused on a superfamily of enzymes named carbonic anhydrases (CAs, EC 4.2.1.1) encoded by the genome of pathogenic and nonpathogenic bacteria [84–86], which are involved in the metabolic balance of the carbon dioxide (CO_2_), bicarbonate (HCO_3_^−^), and protons (H^+^), catalyzing the physiologically crucial reversible reaction of CO_2_ hydration to HCO_3_^−^ and H^+^, according to the following chemical reaction: CO_2_ + H_2_O⇆HCO_3_^−^ + H^+^. Until now, eight CA classes indicated with *α*, *β*, *γ*, *δ*, *ζ*, *η*, *θ*, and *ι* have been described in all kingdoms of living organisms [84, 87–90]. All CA classes strictly conserve the CO_2_ hydration and HCO_3_^−^ dehydration mechanisms, showing an evident convergent evolution phenomenon, having a very low sequence similarity, and different 3D molecular folds and structures [86, 91]. In bacteria, four CA classes (*α*, *β*, *γ*, and *ι*) regulate the CO_2_ and HCO_3_^−^ balance, being the only CA classes encoded by the bacterial genome [84, 91–96].

**Figure 1:**
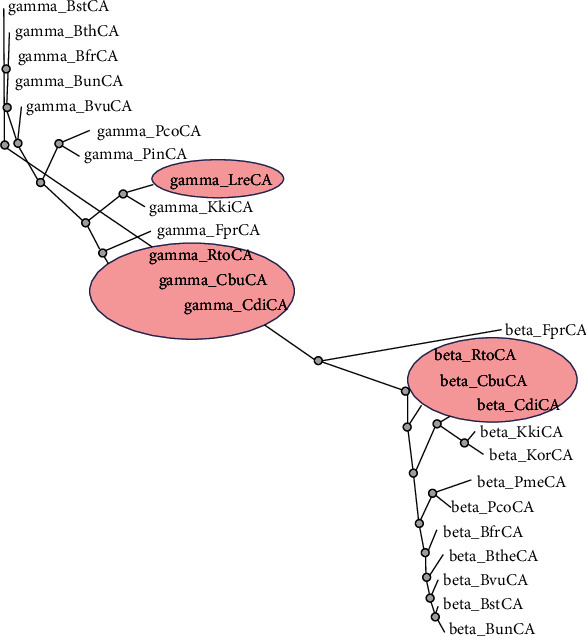
Dendrogram obtained by the phylogenetic analysis carried out on 𝛽- and 𝛾-CAs identified in the genome of the Gram-negative and Gram-positive bacteria indicated in Table 1. The tree was constructed using the program PhyML 3.0. Accession numbers of the amino acid sequences used in the phylogenetic analysis are given in Table 2. Blue circles indicate the Gram-positive bacteria.

Our groups started to explore the genome of both Gram-positive and Gram-negative probiotics for searching CA genes belonging to four different classes (*α*, *β*, *γ*, and *ι*). Some of these bacteria play an essential role in human health, such as *Lactobacillus reuteri*, *Clostridium butyricum*, *Faecalibacterium prausnitzii*, *Akkermansia muciniphila*, *Kingella oralis*, *and K. kingae.* Due to the changes in the microbial composition, others can be considered pathogens for the host, such as *Clostridium difficile*, *Ruminococcus gnavus*, *Prevotella melaninogenica*, and *Bacteroides fragilis.* As reported in Table 1, the considered microorganisms show a CA gene distribution very varied. Some of these bacteria, such as *Lactobacillus reuteri*, *Akkermansia muciniphila*, and *Prevotella intermedia*, show only one CA class, the 𝛾-CA. In contrast, the genome of bacteria, like *Prevotella melaninogenica* and *Kingella oralis*, contained only genes for 𝛽-CAs. At the same time, most of them have 𝛽- and 𝛾-CAs. Again, *Kingella kingae* is the only species among all whose genome is characterized by the presence of the new recently identified class, the 𝜄-class. *Ruminococcus gnavus*, which is generally associated with Crohn's disease, and *Fusobacterium nucleatum*, a bacterium involved in the periodontal disease, are typified by genomes that do not encode for any CA class (Table 1). Intriguingly, the bacteria of the human microbiome here considered resulted in the absence of genes encoding for *α*-CAs. We want to stress that a common feature of the *α*-CAs known to date is the presence of an N-terminal signal peptide, which suggests a periplasmic or extracellular location and a possible physiological role in CO_2_ uptake processes [85, 90]. The lack of *α*-CAs in Gram-negative bacteria could be compensated by the presence of 𝛽- or 𝛾-CAs characterized by a signal peptide, which may have a periplasmic localization and a role similar to that described for the *α*-forms [97]. Interestingly, we constructed a phylogenetic tree to investigate the evolutionary relationship of *β*- and *γ*-CAs identified in the microorganisms reported in Table 1 (Figure 1). As a result, the two CA classes (*β* and *γ*) are grouped in two clusters well separated from each other, indicating how phylogenetically different they are. The 𝛾-CA amino acid sequences can be considered transition amino acid sequences from which the *β*-CAs have originated (Figure 1).

### 4.1. Inhibition of Bacterial CAs

**Figure 2:**
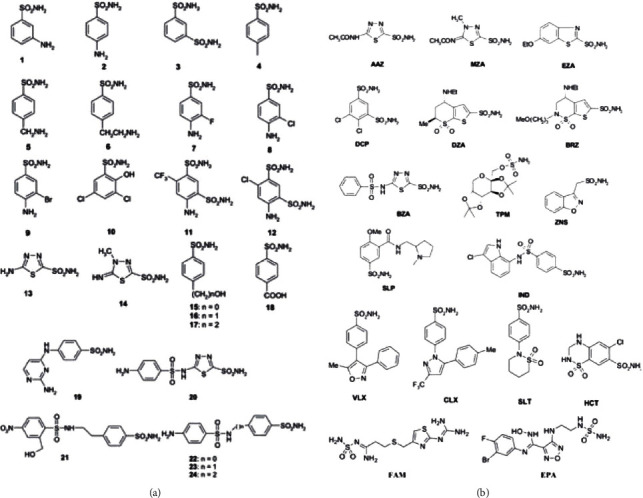
Sulfonamides and their isostere classes (sulfamates and sulfamides) as CAIs. Simple aromatic/heterocyclic derivatives 1-24 (a); clinically used drugs or agents in clinical development (b). Legend: AAZ: acetazolamide; MZA: methazolamide; EZA: ethoxzolamide; DCP: dichlorophenamide; DZA: dorzolamide; BRZ: brinzolamide; BZA: benzolamide; TPM: topiramate; SLT: sulthiame; ZNS: zonisamide; SLP: sulpiride; IND: indisulam; CLX: celecoxib; VLX: valdecoxib; HCT: hydrochlorothiazide; FAM: famotidine; EPA: epacadostat.

CAs, with their activity, continually provide the indispensable CO_2_ and HCO_3_^−^/protons to microbial biosynthetic pathways. Thus, their inhibition might impair the survival of microbes [7]. However, it is important to stress that the inhibition of the human microbiome CAs will not interfere with the human CAs since the mammalian genome encodes only for *α*-CAs, which are phylogenetically and structurally well separated by the bacterial 𝛽- and 𝛾-CAs [7]. Fortunately, many CA inhibitors (CAIs) exist and belong to many chemical classes, such as substituted benzene-sulfonamides, inorganic metal-complexing anions, dithiocarbamates, and carboxylic acids [99–101]. Among them, the sulfonamides shown in Figure 2 are the most potent investigated CA inhibitors (CAIs) (simple derivatives 1-24 and clinically used drugs or agents in clinical development) [87, 98, 102–119]. All of them were shown to also act as CAI primary sulfonamides as these inhibit CAs by binding to the Zn^2+^ ion from the enzyme active site, in a tetrahedral geometry of the metal, whereas the sulfonamide is deprotonated at the SO_2_NH_2_ moiety. The nitrogen atom of the SO_2_NH^−^ group then coordinates the Zn^2+^ ion and participates in a network of H-bonds, which involve conserved amino acid residues (Thr199 and Glu106), which in this way anchor the inhibitor molecule to the enzyme very strongly. This has been demonstrated by X-ray crystallographic studies of many adducts of such sulfonamides with various CA isoforms. The scaffold of the inhibitor (aromatic/heterocyclic moiety) also interacts with amino acid residues from the active site, either in the hydrophilic or within the hydrophobic part of the catalytic cleft.

### 4.2. CA Activators

**Figure 3:**
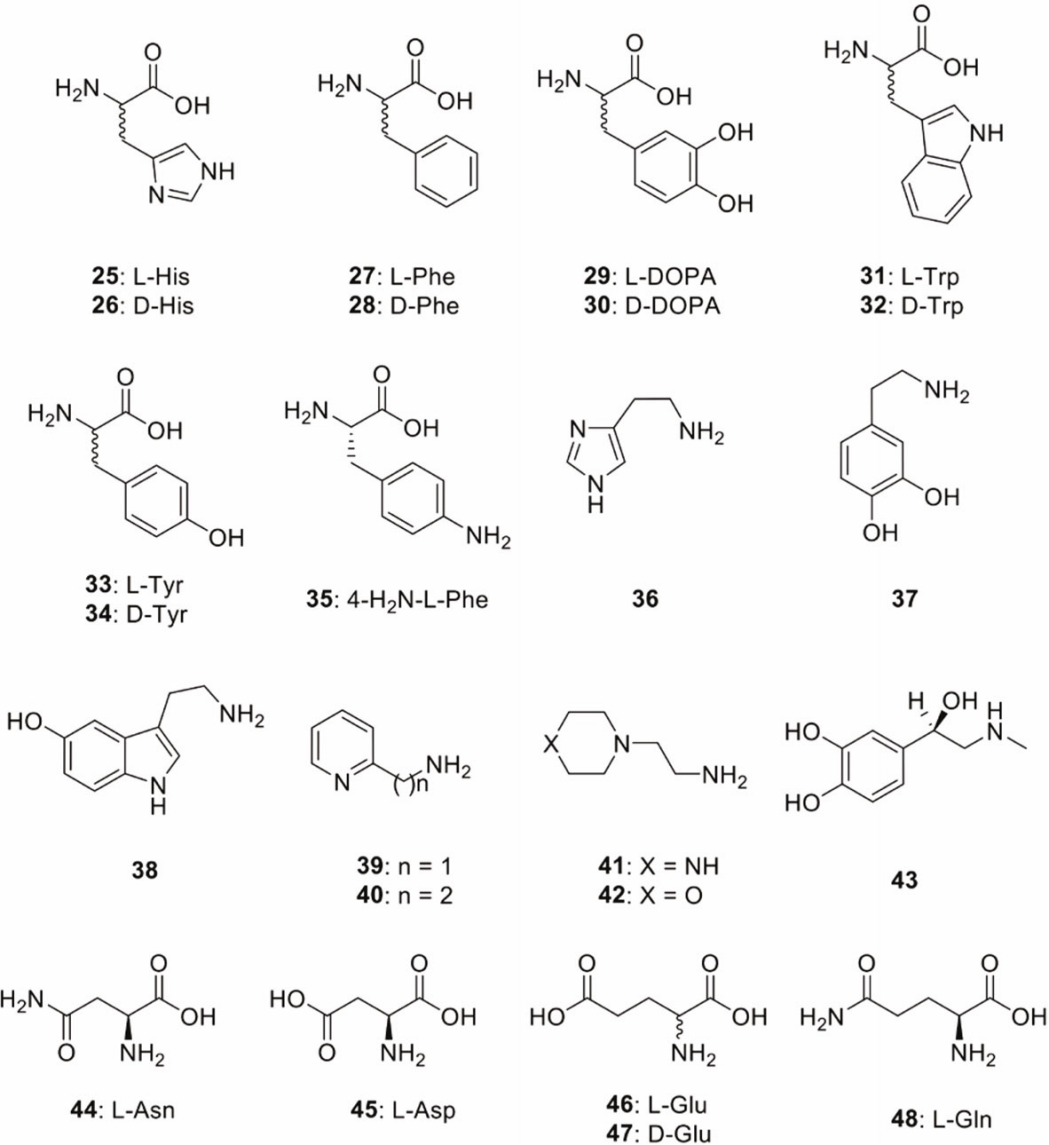
Amino acid and amine activators 25-48 investigated for the activation of several bacterial CAs.

It is possible to assume that the resident gastric microflora responsible for human wellbeing can be reinforced and improved through the increase of the CO_2_ and HCO_3_^−^ produced by enhancing the activity of their bacterial CAs. To accomplish this, the CA activity can be specifically intensified with molecules known as “activators” (CAA), which can bind within the middle-exit part of the enzyme active site. The modulation of the CAs encoded by the human microbiome can be considered a possible new pharmacological treatment since selective CAIs can interfere with the growth of those bacteria responsible for human illness, while the use of selective CAAs could improve the action of the microbiome having a beneficial effect on the host. The CAAs are biogenic amines (histamine, serotonin, and catecholamines—see Figure 3), amino acids, oligopeptides, or small proteins, such as compounds 25-48 shown in Figure 3 [120–122]. The X-ray crystal structure of the human isoforms (hCA I and II) bound to activators, such as histamine, L-/D-histidine, L-/D-phenylalanine, and D-tryptophan, allowed the comprehension of the activation mechanism and the structure-activity relationship governing it [118, 123–129]. CAAs do not influence the binding of CO_2_ to the CA active site but mediate the rate-determining step of the catalysis hurrying the transfer of protons from the active site to the environment. The final result is an overall increase in the catalytic turnover. Thus, the CA activators enhance the *k*_cat_ of the enzyme up to 10^6^ s^−1^ with no effect on *K*_*M*_ [118, 120, 121]. CAAs may have pharmacologic applications in therapy memory, in neurodegenerative diseases (Alzheimer's disease), or in the treatment of genetic CA deficiency syndromes [118, 120, 121]. On the other hand, the activation of bacterial CAs was poorly investigated [130, 131]. For this reason, Vullod et al. and Akdemir et al. investigated the activation profile of the bacterial CAs identified in the genome of the pathogenic and nonpathogenic bacteria to understand better the role of the CAs in the lifecycle and virulence of bacteria [130, 131].

## 5. Conclusion

It is now well established that the gut microbiome in the eubiosis status plays an important role in human physiology by producing numerous molecules and mediators that influence various host functions such as digestion, vitamin production, energy intake, pathogen protection, and immune system maturation/modulation [132]. However, various factors (endogenous and exogenous) can affect the compositional/functional balance of the microbiota, creating a state of dysbiosis, which is the starting point of various gastrointestinal diseases (IBD, CRC, celiac disease, etc.) and not metabolic disorders, immunological dysregulations, mental illnesses, etc. The challenge for modern medicine is to figure out how to reconstruct the gut microbiota to restore it to a healthy balance (eubiosis) and counterbalance its negative involvement in illness onset. This is undoubtedly a difficult challenge as the microbiota is a complex ecosystem that interfaces with an equally complex universe called host/human. The microbiota shaping is, therefore, a delicate process that falls in precision (personalized) medicine [133] and that is currently practiced in different ways, as previously discussed, ranging from the diet (or use of prebiotics, probiotics, and synbiotics) up to the phage therapy and antibiotics, including finally the microbiota fecal transplantation. Because microbiota modulation is a capillary process, and because many microbiota bacteria (both beneficial and pathogenic) have carbonic anhydrases (specifically the four classes *α*, *β*, *γ*, and *ι*), the use of CA inhibitors and activators can open up new therapeutic strategies for many of the diseases related to a maximum microbial dysbiosis, such as the various gastrointestinal disorders and the same colorectal cancer [134], which is currently one of the most common tumors in the world with an age-standardized worldwide incidence of 19.7 and mortality of 8.9 per 100000 person-year [133]. Surgical resection is the golden standard of CRC management. However, according to the clinical and pathological stage and if appropriate, this treatment should be integrated with neoadjuvant and/or adjuvant therapies, such as CA inhibitors and activators that we propose as future integration.

## Figures and Tables

**Table 1:** The genome of Gram-positive and Gram-negative bacteria of the human microbiome encodes for CAs belonging to different classes. Some of the probiotics considered in the present study play an essential role in human health. Others, due to the changes in the microbial composition, can be considered pathogens for the host.

Microorganism	CA class				Disease	Through the production of antimicrobial^a^
	*α*	*β*	*γ*	𝜄		
Gram-positive						
*Lactobacillus reuteri*	−	−	+	−		Intestinal beneficial effects through the production of antimicrobial molecules, organic acids, ethanol, and reuterin, showing antimicrobial activity.
*Clostridium butyricum*	−	+	+	−		Involved positively in antiprogrammed cell death protein-1 (PD-1) treatment, in homeostasis and anti-inflammatory response in inflammatory gut disease.
*Clostridium difficile*	−	+	+	−	Associated with a history of unrelated diarrheal illnesses, such as food poisoning or laxative abuse. Production of toxin, such as enterotoxin and cytotoxin.	
*Ruminococcus gnavus*	−	−	−	−	Associated with Crohn's disease, an inflammatory bowel disease, through the production of an inflammatory polysaccharide.	
*Ruminococcus torques*	−	+	+	−	Bacterial species known to decrease gut barrier integrity.	
Gram-negative						
*Fusobacterium nucleatum*	−	−	−	−	Involved in periodontal disease and colon-rectal cancer (CRC) development.	
*Faecalibacterium prausnitzii* ^∗^	−	+	+	−		Involved positively in antiprogrammed cell death protein-1 (PD-1) treatment. Potentially important role in promoting gut health.
*Akkermansia muciniphila*	−	−	+	−		Intestinal beneficial effects acting as anti-inflammatory.
*Prevotella melaninogenica*	−	+	−	−	Associated with many types of infection, including oral abscesses and infections in the intestinal tract, the female genitalia tract, and the upper and lower respiratory tracts and in the bone marrow. This species interferes with the host inflammatory response.	
*Prevotella copri*	−	+	+	−	Associated with inflammatory diseases, interfering with the host inflammatory response.	
*Prevotella intermedia*	−	−	+	−	Involved in periodontal infections. Interferes with the host inflammatory response.	
*Bacteroides fragilis*	−	+	+	−	Involved in abscess formation and bacteremia.	
*Bacteroides uniformis*	−	+	+	−	Associated with human infections.	
*Bacteroides vulgatus*	−	+	+	−	Associated with human infections.	
*Bacteroides stercoris*	−	+	+	−	Associated with human infections.	
*Bacteroides thetaiotaomicron*	−	+	+	−	Associated with human infections.	
*Kingella oralis*	−	+	−	−	Associated with periodontitis.	Reduces/reduced incidence of oral cavity carcinomas.
*Kingella kingae*	−	+	+	+	Associated with respiratory or urinary tract infections.	Reduces/reduced incidence of oral cavity carcinomas.

^a^[98]. ^∗^*Faecalibacterium prausnitzii* stains like a Gram-negative bacterium but exhibits dermis characteristics that resemble Gram-positive bacteria; ^−^absent; ^**+**^present.

**Table 2:** Microorganisms, CA accession numbers, and protein acronyms of the amino acid sequences used in the phylogenetic analysis.

Microorganism	𝛽-class		𝛾-class	
	*Accession number*	*Acronym*	*Accession number*	*Acronym*
*Lactobacillus reuteri*			WP_163622737.1	gamma_LreCA
*Clostridium butyricum*	ALP89146.1	beta_CbuCA	WP_035762541.1	gamma_CbuCA
*Clostridium difficile*	WP_003423380.1	beta_CdiCA	WP_004454132.1	gamma_CdiCA
*Ruminococcus gnavus*				
*Ruminococcus torques*	WP_144366732.1	beta_RtoCA	CUN19994.1	gamma_RtoCA
*Fusobacterium nucleatum*				
*Faecalibacterium prausnitzii*	WP_158402608.1	beta_FprCA	MBD9046903.1	gamma_FprCA
*Prevotella melaninogenica*	WP_120175219.1	beta_PmeCA		
*Prevotella copri*	WP_203055371.1	beta_PcoCA	WP_089544874.1	gamma_PcoCA
*Prevotella intermedia*			WP_014708543.1	gamma_PinCA
*Bacteroides fragilis*	EEZ25097.1	beta_BfrCA	WP_005814348.1	gamma_BfrCA
*Bacteroides uniformis*	WP_005828510.1	beta_BunCA	WP_118132341.1	gamma_BunCA
*Bacteroides vulgatus*	ABR38061.1	beta_BvuCA	CDF19756.1	gamma_BvuCA
*Bacteroides stercoris*	WP_005652261.1	beta_BstCA	RGZ94434.1	gamma_BstCA
*Bacteroides thetaiotaomicron*	WP_118307725.1	beta_BtheCA	WP_008765423.1	gamma_BthCA
*Kingella oralis*	WP_040558280.1	beta_KorCA		
*Kingella kingae*	WP_019389503.1	beta_KkiCA	WP_019390101.1	gamma_KkiCA
